# Transcatheter Aortic Valve Replacement: The Experience of One Brazilian Health Care Center

**DOI:** 10.21470/1678-9741-2017-0117

**Published:** 2018

**Authors:** Fabiula Schwartz Azevedo, Marcelo Goulart Correa, Débora Holanda Gonçalves Paula, Alex dos Santos Felix, Luciano Herman Juaçaba Belém, Ana Paula Chedid Mendes, Valeria Gonçalves Silva, Bruno Miranda Marques, Andrey José de Oliveira Monteiro, Clara Weksler, Alexandre Siciliano Colafranceschi, Daniel Arthur Barata Kasal

**Affiliations:** 1 Instituto Nacional de Cardiologia (INC), Rio de Janeiro, RJ, Brazil.

**Keywords:** Transcatheter Aortic Valve Replacement, Heart Valve Prosthesis Implantation, Aortic Valve Stenosis/surgery

## Abstract

**Objective:**

Transcatheter aortic valve replacement has been an alternative to invasive treatment for symptomatic severe aortic stenosis in high risk patients. The primary endpoint was 30-day and 1-year mortality from any cause. Secondary endpoints were to compare the clinical and echocardiographic variation pre-and post- transcatheter aortic valve replacement, and the occurrence of complications throughout a 4-year follow-up period.

**Methods:**

This prospective cohort, nestled to a multicenter study (Registro Brasileiro de Implante de Bioprótese por Cateter), describes the experience of a public tertiary center in transcatheter aortic valve replacement. All patients who underwent this procedure between October 2011 and February 2016 were included.

**Results:**

Fifty-eight patients underwent transcatheter aortic valve replacement. The 30-day all-cause mortality was 5.2% (n=3) and after 1 year was 17.2% (n=10). A significant improvement in New York Heart Association functional classification was observed when comparing pre-and post- transcatheter aortic valve replacement (III or IV 84.4% *versus* 5.8%; *P*<0.001). A decline in peak was observed (*P*<0.001) and mean (*P*<0.001) systolic transaortic gradient. The results of peak and mean post-implant transaortic gradient were sustained after one year (*P*=0.29 and *P*=0.36, respectively). Left ventricular ejection fraction did not change significantly during follow-up (*P*=0.41). The most frequent complications were bleeding (28.9%), the need for permanent pacemaker (27.6%) and acute renal injury (20.6%).

**Conclusion:**

Mortality and complications in this study were consistent with worldwide experience. Transcatheter aortic valve replacement had positive clinical and hemodynamic results, when comparing pre-and post-procedure, and the hemodynamic profile of the prosthesis was sustained throughout follow-up.

**Table:** 

Abbreviations, acronyms & symbols	
BNP	= Brain natriuretic peptide
EuroSCORE I	= European System for Cardiac Operative Risk Evaluation
FC	= Functional classification
LVED	= Left ventricle end-diastolic diameter
LVEF	= Left ventricular ejection fraction
TAVR	= Transcatheter aortic valve replacement
TEE	= Transesophageal echocardiogram
TTE	= Transthoracic echocardiogram
VARC-2	= Valve Academic Research Consortium-2

## INTRODUCTION

Symptomatic patients with severe aortic stenosis have limited functionality and survival. For these patients, valve replacement is the treatment of choice. However, the treatment for those with high or prohibitive surgical risk was restricted to medication, as in pre-surgery era. After the advent of transcatheter aortic valve replacement (TAVR) in 2002, symptomatic patients with severe aortic stenosis and high risk have an alternative intervention option, with survival and functional improvement. Its use is nowadays spread worldwide, including in developing countries^[1,2]^. The TAVR experience of public tertiary health care center was described in this prospective cohort.

The primary endpoint was a 1-year mortality due to any cause. Secondary endpoints were to compare functional classification (FC), peak and mean transaortic systolic gradient and left ventricular ejection fraction (LVEF), when comparing before and after the procedure. In addition, the present study aimed to evaluate the complications related to the procedure.

## METHODS

This is a prospective cohort nestled to a multicenter national study (Registro Brasileiro de Implante de Bioprótese por Cateter)^[3]^. The patients included underwent TAVR between December 2011 and January 2016, at a Brazilian public tertiary health care center.

The patient selection protocol started with individuals with severe aortic stenosis or dysfunctional biologic aortic valve being evaluated by a multidisciplinary team (formed by cardiologists, cardiac surgeons and nurses). TAVR was considered as treatment when they presented high or prohibitive surgical risk.

The TAVR was indicated when at least one of the following criteria was present: A-patients were considered as high risk, with European System for Cardiac Operative Risk Evaluation (EuroSCORE I) equal or higher than 15%; or B-the presence of frailty (identified using subjective criteria by the TAVR team, as difficulty in walking and low body weight)^[4]^; or C-porcelain aorta (circumferential calcification of ascending aorta, as seen on computer tomography images) was considered as prohibitive risk for conventional valve replacement surgery.

Eligible patients were evaluated according to New York Heart Association FC, and laboratory parameters before the TAVR procedure. The exams included blood analysis (hemoglobin, creatinine, platelets, brain natriuretic peptide (BNP), electrocardiogram, transthoracic echocardiogram (TTE) and coronariography. Peak and mean transaortic gradients, presence of aortic regurgitation, aortic valve area, left ventricle end-diastolic diameter (LVED) and LVEF (Teicholz method) were evaluated according to American Echocardiography Society^[5]^.

All patients were studied before TAVR by angiotomography images of total aorta, subclavian, femoral and iliac arteries, in order to evaluate eligibility and to choose the site of catheter insertion (femoral, transapical or other), the valve model and size. Exclusion criteria were incompatible size between available prosthesis and the valve ring size (according to angiotomography images), the presence of clot in the ventricle, LVEF < 20% on TTE or estimated survival less than one year.

The valve models used were those available at the time of initial evaluation. The valve size was chosen according to valve ring size on angiotomography. Femoral artery was the preferential access. If not possible, an alternative access was obtained. TAVR was performed under general anesthesia or sedation anesthesia, and accompanied by transesophageal echocardiogram (TEE).

TAVR to treat a dysfunctional biologic prosthetic aortic valve, was called as valve-in-valve procedure and was performed under the same criteria as listed above.

After TAVR procedure, laboratory data was collected in the first 72 hours, electrocardiogram was performed daily, and one TTE was obtained before patient discharge, until 7 days after the procedure.

Functional classification, electrocardiogram and TTE were registered in 30 days, six months, one year and two years after TAVR.

Complications were registered as Valve Academic Research Consortium-2 (VARC-2) definitions^[6]^, as the mortality causes classification. Non identified cause was considered as cardiovascular cause.

### Statistical Analysis

Kolmogorov-Smirnov and Shapiro-Wilk tests were used to check distribution pattern. Continuous variables with normal distribution were presented as mean ± standard deviation, otherwise were presented as median and interquartile range and were submitted to Student t test or Mann-Whitney. Categorical variables were presented as frequencies (number, percentage and confidence interval) and compared through the chi-square and Fisher exact test. Friedman and Cochran Q were used for paired tests and ANOVA to repeated measures. Kaplan Meier survival curve estimated the survival rate free of events in this population and its censored cases were deaths or the last follow-up. Data were analyzed with the R^®^ software 3.1.0 through Extension EZR 1.27 do R Commander 2.1-4. It was assumed 5% alpha error and *P* values ≤ 0.05 were considered significant. This study was registered in the local Research Ethics Committee, according to the Declaration of Helsinki. All participants signed a consent form.

## RESULTS

Fifty-eight patients were treated with TAVR. Baseline characteristics are presented in Tables 1 and 2.

**Table 1:** Baseline clinical profile.

Variable	Population (n=58)
Age in years, mean ± SD	77.8±8.9
Age ≥ 80 years, n (%)	29 (50)
Female, n (%)	36 (62.1)
Body mass index (kg/m^2^), mean ± SD	25.9±5.2
Functional classification II (NYHA), n (%)	9 (15.5)
Functional classification III or IV (NYHA), n (%)	49 (84.9)
Syncope, n (%)	18 (31.1)
Angina, n (%)	23 (39.7)
Atherosclerotic coronary disease, n (%)	33 (56.9)
High blood pressure, n (%)	54 (93.1)
Diabetes mellitus, n (%)	14 (24.4)
Dyslipidemia, n (%)	38 (65.5)
Prior myocardial infarction, n (%)	9 (15.5)
Prior stroke or transient ischemic attack, n (%)	8 (13.8)
High pulmonary pressure, n (%)	11 (19.9)
Carotid artery stenosis, n (%)	15 (25.9)
Peripheral artery disease, n (%)	11 (19.9)
Chronic obstructive pulmonary disease, n (%)	11 (19.9)
Aortic aneurysm, n (%)	5 (8.6)
Porcelain aorta, n (%)	24 (41.4)
Hematological disorders, n (%)	18 (31.1)
Chronic kidney disease, n (%)	42 (72.4)
Creatinine clearance* (mL/min), median [IQR]	47.8 [38.5 - 60.9]
Prior coronary artery bypass graft, n (%)	10 (17.2)
Prior aortic valve replacement, n (%)	7 (12.1)
Percutaneous transluminal coronary angioplasty, n (%)	13 (22.4)
Dysfunctional aortic bioprosthesis, n (%)	7 (12.1)
Pacemaker, n (%)	5 (8.6)
Logistic EuroSCORE (%), median [IQR]	12.7 [8.0 - 20.8]
Sinusal rhythm, n (%)	41 (70.7)
Atrial fibrillation/flutter, n (%)	13 (22.4)
First degree atrioventricular block, n (%)	10 (17.5)
Left bundle branch block, n (%)	7 (12.1)
Right bundle branch block, n (%)	5 (8.6)
Left anterior hemiblock, n (%)	4 (6.9)
Right bundle branch block + left anterior hemiblock, n (%)	5 (8.6)

SD=standard deviation; NYHA=New York Heart Association;

(*) calculated by Cockcroft-Gault formula; IQR=interquartile range; EuroSCORE=European System for Cardiac Operative Risk Evaluation

**Table 2:** Baseline and after transcatheter aortic valve replacement echocardiogram values.

Variable	Baseline		After TAVR*	
	Total**	Value	Total**	Value
LVEF (%), mean ± SD	58	57.4±16	51	59.2±16.4
LVED (mm), median [IQR]	58	52.2 (12.6)	NA	NA
Transaortic peak gradient (mmHg), mean ± SD	56	79.8±22.2	51	20 [16.5-28]
Transaortic mean gradient (mmHg), mean ± SD	57	49.3±15	49	10 [8-14]
Aortic valvular area (cm^2^), mean ± SD	50	0.59±0.19	NA	NA
Severe or moderate aortic regurgitation, n (%)	58	12 (20.7)	55	13 (22.4)
Hb (mg/dL), mean ± SD	58	11.6 (1.5)	57	9.4±1.6
CR (mg/dL), median [IQR]	58	0.9 [0.8-1.3]	57	1.1 [0.9-1.4]
PLT (x10^3^), median [IQR]	58	183.5 [146.2-224.7]	57	133 [109-179]
BNP (pg/mL)	27	316 [125.4 - 906.8]	NA	NA

* Before hospital discharge;

** Total analyzed < 58 = data not available TAVR=Transcatheter aortic valve replacement; LVEF=left ventricle ejection fraction; SD= standard deviation; LVED=left ventricle end-diastolic diameter; IQR=interquartile range; NA=data not available; Hb=hemoglobin; CR=creatinine; PLT=platelets; BNP=Brain natriuretic peptide

General anesthesia was employed in 51 (87.9%) cases. Sedation and local anesthesia were performed in 7 (12.1%) patients and TEE in 53 (91.4%) procedures. The main access was transfemoral (n=47; 81%). The alternative access used were transapical (n=7; 12.1%), transaortic (n=3; 5.2%), and subclavian artery (n=1; 1.7%). Self-expandable valve CoreValve^®^ (Medtronic Inc., Minneapolis, MN, USA) was implanted in 40 (68.9%) patients, balloon-expandable valve SAPIEN-XT^®^ (Edwards Lifesciences, Irvine, CA, USA) in 11 (19%) and Inovare^®^ (Braile Biomedica, São José do Rio Preto, SP, Brazil) in seven (12.1%) cases.

Seven (12.1%) patients had dysfunctional biological aortic prosthesis and the procedures were valve-in-valve. All of them had an Inovare^®^ model implanted surgically via transapical access. Echocardiographic and laboratory data after TAVR are shown in Table 2.

In this cohort, the all-cause mortality was 17.2% (n=10) within the follow-up of 1 year. The mortality within 30 days was 5.2% (n=3) and was considered related to the procedure. Two of them had apical access: their mortality causes were due to life threatening bleeding for both, beside major ischemic stroke for one of them. The third patient that immediately died after the procedure, had atrioventricular block due to contiguous complication.

The late mortality occurred in seven cases, five of them related to cardiovascular causes and the others related to noncardiovascular causes. Among the cardiovascular causes were: one had major ischemic stroke; three of them had sudden death or non-identified cause; and one death was related to the procedure (prosthesis migration and major bleeding). The noncardiovascular related causes were sepsis and rupture of preexistent esophageal varices.

Among the patients that died (n=10), three had moderate or severe aortic regurgitation on the echocardiogram (until seven days after TAVR). They died, each one, 10.7 and 6.5 months (moderate regurgitation) and 1.5 month (severe regurgitation) after the procedure.

Apical access was used to treat dysfunctional bioprosthesis: seven valve-in-valve procedures, all of them using Inovare^®^ model. Among these subgroup, two early deaths were registered (within the first day after TAVR). Both had life threatening bleeding, renal injury and received second valve during the procedure, due to related first valve failure. The first patient had also ventricular perforation and atrioventricular block. The second had also left bundle block and major ischemic stroke. One patient died after the 30^th^ day (late mortality) related to a noncardiovascular cause. The other four valve-in-valve patients did not have any complications.

**Fig. 1:**
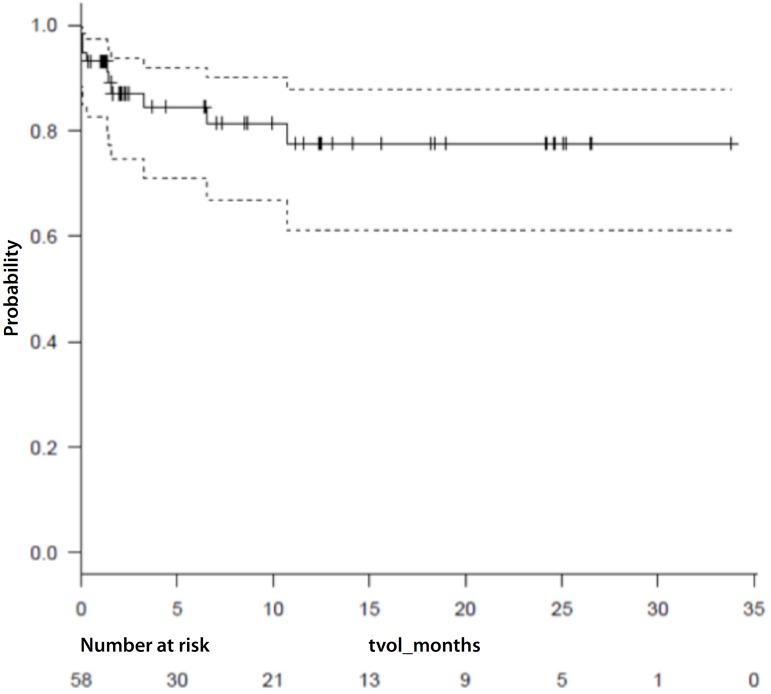
Kaplan-Meier survival curve*. 95% CI - - - - - - Survive ____ + Censored (*) Censored cases represent last follow-up visit.

Survival analysis is represented by Kaplan Meier curve in Figure 1. Mean follow-up time was 6.4 [1.55-12.9] months and maximum 33.8 months in this cohort.

**Fig. 2:**
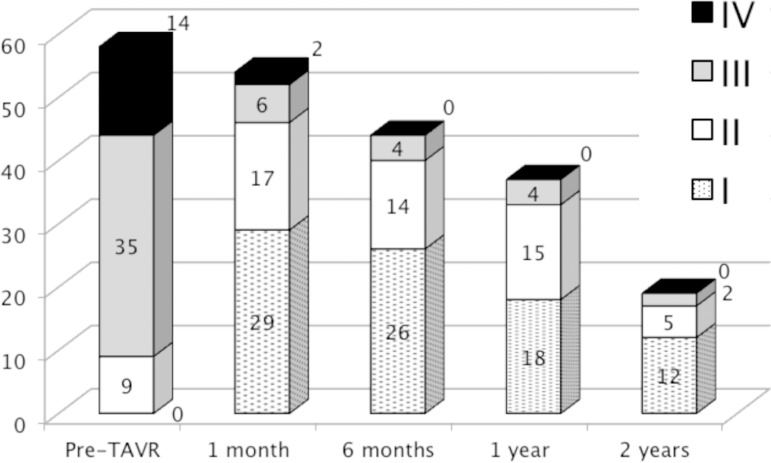
New York Heart Association functional classification during follow-up after transcatheter aortic valve replacement*. * Losses were due to deaths or the last follow-up in timeline.

Clinical evolution of FC is presented in Figure 2. The prevalence of FC III or IV pre-TAVR was 84.5% (n=49 of 58) compared to 30 days that was 14.8% (n=8 of 54; *P*<0.001), six months 9.1% (n=4 of 44; *P*<0.001); one year 10.8% (n=4 of 37; *P*<0.001); and two years 10.5% (n=2 of 19; *P*<0.001).

**Fig. 3:**
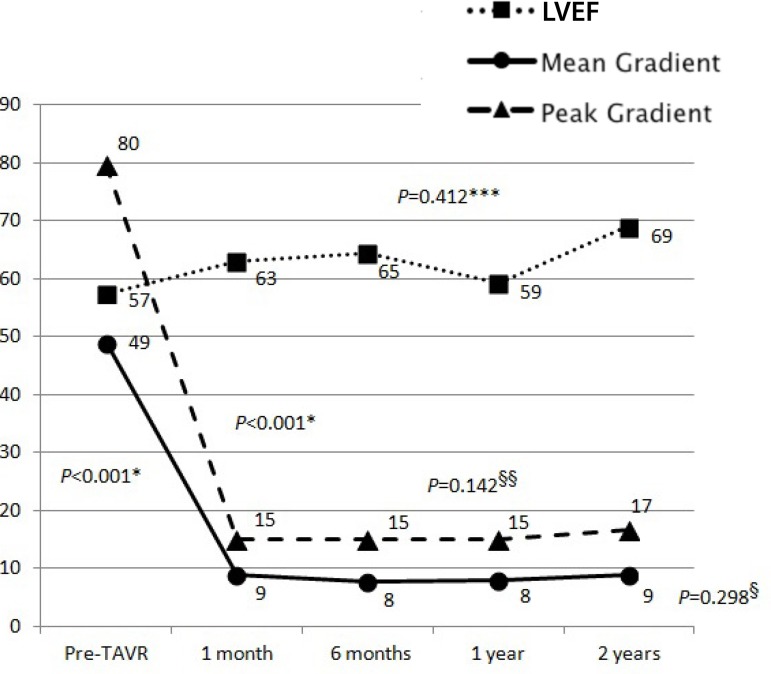
Left ventricular ejection fraction, transaortic peak, and mean gradient evolution (mmHg). LVEF=Left ventricle ejection fraction; Transaortic (*) Peak and (**) mean gradients variability before and after TAVR, before hospital discharge. Transaortic (§) mean and (§§) peak gradient after TAVR

Echocardiographic evolution by transaortic gradient and LVEF analysis before and after TAVR are shown in Figure 3. Complications are presented in Table 3.

**Table 3:** Complications during the 4-year follow-up period according to Valve Academic Research Consortium-2.

Variable	Value
Stroke, n (%)	4 (6.9)
Myocardial infarction, n (%)	1 (1.7)
Hemorrhagic complications, n (%)	17 (29.3)
Minor bleeding, n (%)	7 (12.1)
Major bleeding, n (%)	4 (6.9)
Life threatening bleeding, n (%)	6 (10.3)
Vascular complication, n (%)	5 (8.6)
Minor vascular complication, n (%)	3 (5.2)
Major vascular complication, n (%)	2 (3.4)
Acute kidney injury, n (%)	12 (20.6)
Stage 1, n (%)	5 (8.6)
Stage 2, n (%)	3 (5.2)
Stage 3, n (%)	2 (3.4)
Pacemaker, n (%)	16 (27.6)
Periprosthetic regurgitation ≥ moderate, n (%)	8 (14.8)*
Second prosthesis implantation, n (%)	7 (12.1)
Cardiovascular mortality, n (%)	8 (13.8)

* This data was analyzed from available echocardiogram data after TAVR (until seven days after the procedure) of 54 patients.

## DISCUSSION

This prospective cohort describes the experience of a public tertiary center in TAVR.

The small population and the single center analysis are limitations of this study. Frailty was considered according to subjective impressions of a multidisciplinary team and may interfere on the reproducibility of selection criteria.

Mean age in this cohort was similar to main published registries. Mean EuroSCORE was inferior comparing to previous studies. However, porcelain aorta was more prevalent in this population^[7-9]^. As porcelain aorta is a formal surgery contraindication and it could have impacted on EuroSCORE profile of this population^[7]^.

General anesthesia was preferred over sedation and local anesthesia. Although recent published studies reveal the opposite, this scenario is similar to publications of initial experiences^[10]^.

As published in Brazilian^[3]^ and international literature^[11,12]^, transfemoral was the main used access. Apical access was used in all valve-in-valve procedure (seven cases) and contributed 20% (two cases) to all-mortality in this cohort.

In cases of dysfunctional aortic bioprosthesis, conventional valve replacement is associated with higher risk compared to the first cardiac surgery and may contraindicate the surgery intervention^[13]^. Valve-in-valve has shown as a safe alternative and has shown adequate hemodynamic performance^[14,15]^. In this cohort, valve-in-valve procedures were not analyzed apart and can interfere on results, mainly on transaortic gradients.

Mortality was consistent with literature. Brazilian registry^[3]^ had 30 days and one year mortality of 9.1% and 21.5%, respectively. Data presented in SOURCE registry^[16]^ was 6.3% e 10.3% (transfemoral and transapical access, respectively); on ADVANCE trial^[17]^ was 4.5%; on FRANCE trial^[7]^, 12.7%; and FRANCE 2 trial^[18]^, 9.7%; survival analysis after one year in PARTNER registry^[8]^ was 76.9%, in SOURCE^[16]^ was 76.1%, in FRANCE 2^[18]^ 81.6%, and Figulla et al.^[19]^ showed 75.9%.

Aortic regurgitation of at least moderate level in this cohort was 14.81%. Although TEE had been performed during most procedures^[20,21]^, these data were not available for analysis. The use of TTE data done until seven days after the procedure as the first echocardiogram after TAVR can limit comparisons among other studies echocardiogram data, since TEE or TTE done in the operating room is the standard in literature. To avoid further data interference, two cases without echocardiogram data before the discharge were excluded from the population analyzed.

Recently, the new generation of the balloon-expandable valve system has shown less occurrence of aortic regurgitation and pacemaker implantation^[22]^. This cohort had clinical endpoints and complications defined by VARC-2^[23]^. Accordingly, results should be carefully compared to the literature, since the same criteria and definitions should have been used for comparisons.

Hemorrhagic complications and pacemaker implantation were the main complications in this study. Comparing to literature, hemorrhagic complications were more frequent and acute renal injury occurrence was less frequent in this cohort, although there is a large variation of criteria used among different studies. Pacemaker implantation was similar to the literature, consistent to the registries which indicate that this complication is more frequent among the self-expandable valve model CoreValve^®^ model^[24-26]^. In this cohort, this model was used in almost 70% of the procedures.

The post-TAVR echocardiogram (until seven days after the procedure) data were not available in four patients. Two of them died during the procedure. The other two had post-TAVR echocardiogram registries 30 days after the procedure (one with moderate aortic regurgitation and the other without aortic regurgitation). Thus, among the 54 patients analyzed, one (1.85%) had severe, seven (12.96%) had moderate, twenty-three (42.59%) had mild and twenty-three (42.59%) did not have aortic regurgitation on the echocardiogram before hospital discharge.

Also consistent with literature, significant, lasting clinical and echocardiographic improvement was registered^[27]^. FC improvement could be seen since the first follow-up appointment, changing the profile of III and IV predominance to I and II. Transaortic gradient, peak and mean, fall immediately, maintaining this pattern during follow-up, as in previous studies^[21,26]^. LVEF did not change significantly in this study, however, published studies show improvement in dilated cardiomyopathy after TAVR^[26,27]^.

## CONCLUSION

Mortality and complications in this study were consistent with literature. The more frequent complications in this cohort were hemorrhagic complications and pacemaker implantation. TAVR had positive clinical and hemodynamic results for this population, when comparing pre-and post-procedure. The prosthesis hemodynamic profile was sustained and LVEF did not change significantly during follow-up.

**Table:** 

Authors' roles & responsibilities	
FSA	Substantial contributions to the conception or design of the work; or the acquisition, analysis, or interpretation of data for the work; drafting the work or revising it critically for important intellectual content; final approval of the version to be published
MGC	Substantial contributions to the conception or design of the work; or the acquisition, analysis, or interpretation of data for the work; final approval of the version to be published
DHGP	Substantial contributions to the conception or design of the work; or the acquisition, analysis, or interpretation of data for the work; final approval of the version to be published
ASF	Agreement to be accountable for all aspects of the work in ensuring that questions related to the accuracy or integrity of any part of the work are appropriately investigated and resolved; final approval of the version to be published
LHJB	Agreement to be accountable for all aspects of the work in ensuring that questions related to the accuracy or integrity of any part of the work are appropriately investigated and resolved; final approval of the version to be published
APCM	Agreement to be accountable for all aspects of the work in ensuring that questions related to the accuracy or integrity of any part of the work are appropriately investigated and resolved; final approval of the version to be published
VGS	Agreement to be accountable for all aspects of the work in ensuring that questions related to the accuracy or integrity of any part of the work are appropriately investigated and resolved; final approval of the version to be published
BMM	Substantial contributions to the conception or design of the work; or the acquisition, analysis, or interpretation of data for the work; final approval of the version to be published
AJOM	Substantial contributions to the conception or design of the work; or the acquisition, analysis, or interpretation of data for the work; final approval of the version to be published
CW	Agreement to be accountable for all aspects of the work in ensuring that questions related to the accuracy or integrity of any part of the work are appropriately investigated and resolved; final approval of the version to be published
ASC	Drafting the work or revising it critically for important intellectual content; final approval of the version to be published
DABK	Drafting the work or revising it critically for important intellectual content; final approval of the version to be published
